# Targeted Oral Fixed-Dose Combination of Amphotericin B‑Miltefosine for Visceral Leishmaniasis

**DOI:** 10.1021/acs.molpharmaceut.4c01133

**Published:** 2025-02-17

**Authors:** Raquel Fernández-García, David Walsh, Peter O’Connell, Luiz Felipe D. Passero, Jéssica A. de Jesus, Marcia Dalastra Laurenti, María Auxiliadora Dea-Ayuela, M. Paloma Ballesteros, Aikaterini Lalatsa, Francisco Bolás-Fernández, Anne Marie Healy, Dolores R. Serrano

**Affiliations:** † Departamento de Farmacia Galénica y Tecnología Alimentaria, Facultad de Farmacia, 16734 Universidad Complutense de Madrid. Plaza de Ramón y Cajal, s/n, 28040 Madrid, Spain; ‡ School of Pharmacy and Pharmaceutical Sciences, 8809 Trinity College Dublin, Dublin 2, Ireland; § Institute of Biosciences, 28108 São Paulo State University (UNESP), Praça Infante Dom Henrique, s/n, São Vicente, São Paulo 11330-900, Brazil; ∥ Institute for Advanced Studies of Ocean (IEAMAR), São Paulo State University (UNESP), Rua João Francisco Bensdorp, 1178. São Vicente, São Paulo 11350-011, Brazil; ⊥ Laboratório de Patologia das Moléstias Infecciosas (LIM/50), Faculdade de Medicina, Universidade de São Paulo, Avenida Dr. Arnaldo, 455, Cerqueira César, São Paulo 01246903, Brazil; # Departamento de Farmacia, Facultad de Ciencias de la Salud, Universidad Cardenal Herrera-CEU, Carrer Santiago Ramón y Cajal, s/n., 46113 Valencia, Spain; ¶ Instituto Universitario de Farmacia Industrial, Facultad de Farmacia, 16734 Universidad Complutense de Madrid, Plaza de Ramón y Cajal, s/n., 28040 Madrid, Spain; ∇ CRUK Formulation Unit, School of Pharmacy and Biomedical Sciences, University of Strathclyde, John Arbuthnot Building, Robertson Wing, 161 Cathedral Street, Glasgow G4 0RE, U.K.; ○ Departamento de Microbiología y Parasitología, Facultad de Farmacia, Universidad Complutense de Madrid, Plaza de Ramón y Cajal, s/n., 28040 Madrid, Spain

**Keywords:** oral delivery, amphotericin B, fixed-dose combination, miltefosine, coating, visceral leishmaniasis

## Abstract

The incidence of visceral leishmaniasis (VL) remains a significant health threat in endemic countries. Fixed-dose combination (FDC) of amphotericin B (AmB) and miltefosine (MLT) is a promising strategy for treating VL, but the parenteral administration of AmB leads to severe side effects, limiting its use in clinical practice. Here, we developed novel FDC granules combining AmB in the core with a MLT coating using wet granulation followed by the fluidized bed technology. The granules maintained the crystalline structure of AmB throughout manufacturing, achieving an AmB loading of ∼20%. The MLT coating layer effectively sustained AmB release from 3 to 24 h following Korsmeyer–Peppas kinetics. The formulation demonstrated remarkable stability, maintaining >90% drug content for over a year at both 4 °C and room temperature under desiccated conditions. In vivo efficacy studies in Leishmania infantum *-*infected hamsters showed 65–80% reduction in parasite burden in spleen and liver, respectively, suggesting potential as an oral alternative to current VL treatments. Uncoated and coated granules demonstrated comparable performance in key aspects, including *in vivo* efficacy and long-term stability.

## Graphical abstract

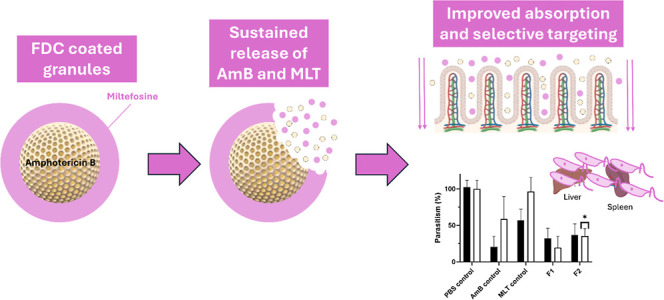

## 1 Introduction

Visceral leishmaniasis (VL), also referred to as kala-azar or black fever, is the most severe form of leishmaniasis.^1^ VL is a potentially fatal disease caused by protozoan parasites of the Leishmania genus, primarily Leishmania donovani and Leishmania infantum.^2^ These parasites target internal organs, such as the liver, spleen, and bone marrow. Symptoms include fever, weight loss, fatigue, anemia, and hepatosplenomegaly.^2^ Despite control efforts reducing incidence in some areas, VL remains a significant health threat in East Africa, India, the Mediterranean basin, China, the Middle East, and South America. The disease is particularly concerning in patients coinfected with the human immunodeficiency virus (HIV).^3–5^ To address this situation, a strategy that has been gaining attention in recent years is the coadministration of multiple drugs with different mechanisms of action. This approach offers several advantages, including reduced adverse effects due to lower required doses to achieve the same efficacy as monotherapy, increased overall efficacy, and improved patient compliance as the treatment duration is shortened.^6–9^

Fixed-dose combination (FDC) therapy has emerged as a promising approach to treating several diseases and infections, including HIV, diabetes, dyslipidaemia, hypertension, and gynecological disorders.^10–12^ For leishmaniasis, the most effective joint treatment combines parenteral amphotericin B (AmB) with oral miltefosine (MLT), showing significantly higher cure rates than monotherapy.^4,13^ However, the utilization of this combination therapy is hampered by several factors. The poor aqueous solubility of AmB limits its oral bioavailability (0.2–0.5%), requiring intravenous administration and hospitalization.^14,15^ Several oral AmB formulations are under development based on nanoparticles, microemulsions, and cochleates, but full parasite eradication in target tissues, such as the liver and spleen, still remains a challenge.^16,17^ This poses a particular challenge in developing countries, where healthcare resources are often limited. On the other hand, MLT, while orally bioavailable, brings several concerns, including drug resistance, adverse effects, and teratogenicity.^18–20^ To address these issues, developing an oral FDC product for VL treatment could revolutionize clinical practice. The antiparasitic activity of AmB stems from its ability to bind to ergosterol in plasma membranes, forming pores that lead to a leakage of ions from the cytoplasm of parasitic cells, resulting in apoptosis.^21–23^ MLT, however, acts as a lipid biosynthesis inhibitor.^24,25^ Formulating AmB as part of an oral FDC product could potentially enhance its overall antiparasitic effect through complementary mechanisms of action. The pore-forming activity of AmB may facilitate increased cellular uptake of MLT, while the disruption of lipid biosynthesis caused by MLT could potentially increase membrane fluidity, enhancing the pore-forming ability of AmB. Additionally, the oral FDC formulation might improve the bioavailability of AmB, addressing one of its main limitations as a parenteral drug.^9^ This strategy represents a promising approach to current treatment limitations and an opportunity to address an unmet clinical need in VL therapy.

Wet granulation is a widely used technique for producing micron-sized granules. This process enhances the physicochemical properties of powder mixtures, particularly flow characteristics and compatibility, which are crucial for tablet manufacturing.^26^ Although conceptually simple, involving the blending of drugs with excipients, wet granulation requires precise control to achieve optimal results.^26^ Granules can serve not only as an intermediate step in tablet production but also as a final dosage form.^27^ The key to successful granulation lies in achieving an optimal balance between the active pharmaceutical ingredient and the selected excipients.^26^ Through wet granulation, immediate-release dosage forms can be formulated, allowing for rapid drug release and fast onset of action following absorption in the gastrointestinal tract.^28^ After drying and hardening, the granules can undergo further processing through spray-coating in a fluidized bed system. This additional step can alter the external properties of the granules without affecting their internal structure. Coatings serve multiple purposes, including protection against chemical and physical degradation of drugs and modification of drug release profiles.^28^ FDC granules are advancing the pharmaceutical technology field in which drugs can be either incorporated in the coating layer or the core protected from moisture and degradation.^9^

The hypothesis that drives this work relies on the use of FDC combining AmB and MLT in a single oral dosage form that could lead to a more effective and safer antileishmanial treatment compared to AmB-based monotherapy, while avoiding adverse effects associated with parenteral administration. The synergy of their different mechanisms of action may result in an additive effect, potentially allowing for lower doses to elicit the same pharmacological effect. In this work, an advanced oral controlled-release FDC formulation has been developed based on water-dispersible granules with an AmB-containing core and an MLT coating layer. Active excipients were carefully selected to enhance the oral bioavailability of AmB and minimize adverse effects in the gastrointestinal tract. The rationale behind this formulation lies in the poor aqueous stability in gastric acidic media of AmB and hence, granules were engineered to place AmB in the core protected by a coating layer containing MLT. Comprehensive studies were conducted to understand the physicochemical properties of the FDC formulation and *in vivo* performance to assess AmB bioavailability, efficacy, and safety profiles. This approach aims to overcome the limitations of current antileishmanial therapies by combining the benefits of two established drugs in a novel, patient-friendly formulation.

## 2 Materials and Methods

### 2.1 Materials

AmB was sourced from North China Pharmaceutical Huasheng Co (Hebei, China) and MLT monohydrate from Xi’an Lyphar Biotech Co., Ltd. (Xian City, China). Inulin Frutafit HD (average degree of polymerization: 10) was provided by Sensus (Roosendaal, The Netherlands). Microcrystalline cellulose (MCC) Avicel PH-101 was supplied by FMC Corporation (Pennsylvania, USA). Chitosan (Mw: 100 kDa) was purchased from Guinama S.L.U. (Valencia, Spain). Sodium deoxycholate (NaDC), ethanol, and dichloromethane (DCM) were supplied from Sigma-Aldrich Chemie GmbH (Buchs, Switzerland). Polyvinyl caprolactam-polyvinyl acetate-polyethylene glycol graft copolymer (Soluplus) and polyvinylpyrrolidone (PVP) Kollidon 17PF (K17) were a gift from BASF SE (Ludwigshafen, Germany). Sodium salts (Na_2_HPO_4_ and NaH_2_PO_4_, analytical grade) were purchased from Panreac Química S.A.U. (Barcelona, Spain). A nonionic polyoxyethylene–polyoxypropylene block copolymer (Poloxamer 188, Pluronic F68) was purchased from Thermo Fisher Scientific Inc. (Massachusetts, USA). Hydroxypropyl methylcellulose–acetate succinate (HPMC–AS, pharmaceutical grade) Aqoat AS-HG was donated by Shin-Etsu Chemical Co., Ltd. (Tokyo, Japan). Solvents of HPLC grade solvents were used. All other chemicals were of reagent grade and were used without further purification.

### 2.2 Methods

#### 2.2.1 Preparation of Amphotericin B–Miltefosine Fixed-Dose Combination Granules

FDC granules were prepared using a two-step process of wet granulation followed by spray-coating, as previously described.^9^ The composition of the granule core and the theoretical amounts of each component are detailed in Table 1.

**Table 1:** Composition of FDC Granule Core^a^

component	weight percentage (%)
AmB	25.00
inulin	17.64
MCC	19.60
chitosan	10.00
NaDC	6.83
Soluplus	13.64
Na_2_HPO_4_	5.00
NaH_2_PO_4_	2.25

a Key: AmB, MCC, and NaDC.

The listed ingredients were accurately weighed and blended using a mortar and a pestle. A 2.5% w/v aqueous solution of PVP K17 was added dropwise as a binder until a solid wet mass formed. The wet mass was passed through a series of sieves (Endecotts Ltd., London, United Kingdom) (1 mm, 710, 500, 350, and 149 μm) to obtain granules of different sizes. The granules were dried overnight at room temperature, protected from light. The largest mass fraction (>500 μm) was separated for spray-coating to minimize attrition.

The spray-coating process was conducted using a Mini-Glatt fluidized bed coater equipped with a Wurster insert (Glatt, Binzen, Germany). Coating solutions were prepared with a 1:2 MLT/excipient weight ratio. Two formulations were manufactured using either Soluplus alone or a mixture of HPMC–AS and Poloxamer 188 (2:1 w/w) as excipients. The drug–polymer mixture was dissolved in 170 mL of ethanol/DCM (1:1 v/v) with a drug concentration of 8.4 mg/mL. The coating parameters were set as follows: 40 °C inlet temperature, 0.5 mm nozzle diameter, 3.8 g/min spray rate, 25 m^3^/h nitrogen flow rate, and 0.8 bar atomization pressure.

#### 2.2.2 Physicochemical Characterization of Fixed-Dose Combination-Coated Granules

##### 2.2.2.1A mphotericin B Content

AmB content within the granules was determined by dispersing 10 mg of granules (*n* = 3) in 2 mL of dimethyl sulfoxide (DMSO) (Scharlab S.L., Barcelona, Spain). The resulting dispersion was filtered through 0.45 μm PTFE filters (Thermo Fisher Scientific Inc., Massachusetts, USA) and appropriately diluted with methanol for HPLC analysis. A Jasco HPLC (Jasco Co., Tokyo, Japan) was used, comprising a DG-2080-53 3-line degasser, a LG-2080-02 ternary gradient unit, a PU-1580 ternary pump, an UV-1575 intelligent UV/vis detector, and a AS-2050 Plus intelligent autosampler. Chromatographic separation was achieved using a BDS Hypersil C18, 5 μm (200 × 4.6 mm) column (Thermo Fisher Scientific Inc., Massachusetts, USA). The mobile phase consisted of acetonitrile/acetic acid/water (52:4.3:43.7, v/v/v) filtered through a 0.45 μm Supor-450 membrane filter with a diameter of 47 mm (Pall Co., Michigan, USA). An isocratic elution method was employed for elution at room temperature at a flow rate of 1 mL/min. The injection volume was 100 μL, and UV detection was performed at 406 nm. Data analysis was conducted using Borwin software.^29^

##### 2.2.2.2 Particle Size Distribution

Particle size distribution (PSD) was determined using a Mastersizer 2000 instrument (Malvern Panalytical Ltd., Malvern, United Kingdom) through laser diffraction. Measurements were performed in triplicate. The instrument was equipped with a Scirocco dry powder feeder operating at 1 bar pressure and a vibration feed rate of 50%. To evaluate the thickness of the coating layers, the median particle size (*D*_50_) values were obtained for both the uncoated and coated formulations. The coating thickness was then calculated using eq 1:^30^

1 $$thickness\;\left(\mu m\right)=\frac{D_{50}\;after\;coating-D_{50}\;before\;coating}{2}$$

##### 2.2.2.3 Surface Area Analysis

Surface area measurements were conducted using the Brunauer–Emmett–Teller (BET) isotherm method with a Micromeritics Gemini 2385c surface area and pore size analyzer (Micromeritics Instrument Corp., Georgia, USA). The BET multiple-point method was employed using nitrogen adsorption to determine surface area. Six data points were obtained within the relative pressure (*P*/*P*_0_) range of 0.05–0.3. Before analysis, samples were prepared by purging under nitrogen overnight at 25 °C. Each result represented the average of three measurements.^31^

##### 2.2.2.4 Scanning Electron Microscopy

The morphology of the formulations was examined by using scanning electron microscopy (SEM). A JSM 6335F (Jeol Ltd., Tokyo, Japan) equipped with a secondary electron detector was employed, providing image resolutions of 1.5 nm at 15 kV and 5 nm at 1 kV. To visualize the surface, the coating layer, and the core of the coated formulations, granules were halved using a sharp blade. The samples were then mounted on aluminum pins using carbon tabs and sputter-coated with gold prior to imaging.

##### 2.2.2.5 Thermogravimetric Analysis (TGA)

Water content was determined using thermogravimetric analysis (TGA) with a TGA Q50 measuring module (TA Instruments, Delaware, USA). Samples were analyzed in triplicate (*n* = 3) using aluminum pans, with nitrogen as the purge gas. The temperature range for analysis was 50–200 °C, with a heating rate of 10 °C/min. Data analysis was performed using TA Universal Analysis software version 4.5A (TA Instruments, Delaware, USA).

##### 2.2.2.6 Modulated Temperature Differential Scanning Calorimetry

Modulated temperature-differential scanning calorimetry (MT-DSC) analysis was conducted using a DSC Q200 instrument (TA Instruments, Delaware, USA) with nitrogen as the purge gas. Samples weighing 2–3 mg were placed in aluminum pans and analyzed over a temperature range of 0–200 °C. The modulation rate was set at 0.8 °C every 60 s, with a scanning rate of 5 °C/min. TA Universal Analysis software version 4.5A (TA Instruments, Delaware, USA) was used for data processing. The instrument was calibrated using an indium as a standard. Melting event temperatures (*n* = 3) were reported as onset temperatures.

##### 2.2.2.7 Fourier-Transform Infrared Spectroscopy

Fourier-transform infrared spectroscopy (FTIR) spectroscopy analysis was performed using a Spectrum 1 FT-IR spectrometer (PerkinElmer Inc., Massachusetts, USA) equipped with a universal attenuated total reflectance accessory and a ZnSe crystal. Spectra were collected in sextuplicate (*n* = 6) over the range of 600–4000 cm^–1^. Data normalization was conducted using Spectragryph software version 1.2.9 (The Spectroscopy Ninja, Berchtesgaden, Germany).

##### 2.2.2.8 Powder X-ray Diffraction

Powder X-ray diffraction (PXRD) analysis was performed in triplicate using a Miniflex II diffractometer (Rigaku Co., Tokyo, Japan). The instrument employed Ni-filter Cu Kα radiation with a wavelength of 1.54 Å. Operating conditions were set at a 30 kV tube voltage and 15 mA tube current. PXRD patterns were recorded over a 2θ range from 5° to 40° at a step scan rate of 0.05°.

##### 2.2.2.9 Dynamic Vapor Sorption

Vapor sorption experiments were conducted using a dynamic vapor sorption (DVS) Advantage-1 automated gravimetric sorption analyzer (Surface Measurement Systems Ltd., London, United Kingdom) at 25 ± 0.1 °C, with water as the probe vapor. Samples (15–20 mg) were initially dried at 0% relative humidity (RH) for 1 h. The RH was then cycled from 10% to 90% for sorption and reversed for desorption. The RH was changed only after the sample mass reached equilibrium, defined as d*m*/dt ≤ 0.002 mg/min over 10 min. Two complete sorption–desorption cycles were recorded for each sample.^32^ Following DVS analysis, samples were evaluated by PXRD.

#### 2.2.3 Dissolution Profile of Amphotericin B

Dissolution studies were conducted using simulated gastric fluid (SGF) and simulated intestinal fluid (SIF). SGF (500 mL) without enzymes was prepared using HCl and deionized water, adjusting the pH to 1.2.^33^ An Erweka DT80 dissolution apparatus (Erweka GmbH, Hessen, Germany) was used, while the medium was heated to 37 °C for 1 h to simulate physiological temperature. Granules (25 mg) were added to each vessel, and dissolution was performed using paddles at 100 rpm under sink conditions in two steps. First, SGF was used for 30 min, followed by the addition of 400 mL of SIF supplemented with 0.5% (w/v) sodium dodecyl sulfate (Thermo Fisher Scientific, Massachusetts; USA) to ensure AmB dissolution under sink conditions. The pH was then adjusted to 6.8 using 2.5 mL of a 30% (w/v) NaOH solution. Samples (5 mL) were withdrawn without media replacement at several time points (5, 10, 15, 30, 45, 60, 90, 120, 150, 180, 240, 360, and 1440 min), centrifuged at 5000 rpm for 5 min, and the supernatant was analyzed using the previously described HPLC method. All dissolution media were prepared according to Pharmacopoeia standards, and studies were performed in triplicate.^33^ The dissolution profile was fitted to different release kinetics models, including zero order (constant dissolution regardless of concentration), first order (release depending on concentration), Higuchi (for drug release from an insoluble matrix based on Fickian diffusion), Korsmeyer–Peppas (for polymeric systems with multiple release mechanisms simultaneously), and Hixson–Crowell (for formulations with uniform particle size).^34–36^ The dissolution profile of MLT was not evaluated considering its high aqueous solubility (≥2.5 mg/mL).^25^

#### 2.2.4 Accelerated Predictive Stability Studies

Prior to accelerated stability testing, all formulations were stored in a desiccator containing silica gel at 4 ± 1.21 °C and 11.01 ± 1.64% RH. The Cuspor Aging System was employed for aging each formulation. Humidity capsules were placed in Cuspor chambers and pre-equilibrated at the selected temperature to ensure stable RH conditions before introducing the samples.^37^ Approximately, 5 mg of each sample was weighed into uncapped HPLC vials and placed in the stability chambers. Granules were exposed to various temperature and RH conditions: 50 °C/10% RH, 50 °C/50% RH, 60 °C/75% RH, 70 °C/10% RH, and 70 °C/50% RH (Table 2). At predetermined time points, samples were collected, dissolved in DMSO, further diluted with mobile phase, and analyzed using the previously described HPLC method.

**Table 2:** Accelerated Predictive Stability Studies (APS) Time Points and Storage Conditions of Granules

time (days)	temperature (°C)	RH (%)
1	60	75
	70	10
		50
2	60	75
3	50	50
	70	10
		50
5	60	75
7	50	10
		50
	60	75
	70	10
		50
10	70	10
		50
14	50	10
		50
21	50	10

Stability modeling was conducted to analyze the degradation kinetics of AmB. The degradation data were fitted to various models, including zero order, first order, second order, Avrami, and diffusion models. To comprehensively understand the combined effects of temperature and RH on AmB, a humidity-corrected Arrhenius equation was used (eq 2)^38,39^

2 $$\ln K=\ln A-\frac{E_{a}}{RT}+B\left(RH\right)$$

where *K* was the degradation constant, *A* was the collision frequency, *E*_a_ was the activation energy, *R* was the gas constant, *T* was the absolute temperature, *B* was the humidity sensitivity factor, and RH was the relative humidity. The *B* term was calculated using the following equation (eq 3)­

3 $$B\left(RH\right)=\frac{\mathrm{Ln}\left(\frac{k_{1}}{k_{2}}\right)}{{RH}_{1}-{RH}_{2}}$$

where *k*_1_ and *k*_2_ were the degradation constants calculated at the same temperature but different relative humidities, RH_1_ and RH_2_, respectively. An averaged *B* term calculated at different temperatures was used. Long-term stability studies were performed at 25 °C/10% RH and 4 °C/10% RH.

#### 2.2.5 In Vitro Antiparasitic Activity

Granules were also evaluated in terms of antiparasitic activity against L. donovani and L. infantum according to previously described protocols.^40,41^ The experiment was carried out in promastigote forms of Leishmania. Parasites were cultured in Schneider’s insect medium (Merck KGaA, Darmstadt, Germany) at 26 °C and supplemented with heat-inactivated fetal bovine serum (FBS) (Merck KGaA, Darmstadt, Germany), penicillin, and streptomycin at a concentration of 100 IU/mL and 100 μg/mL, respectively. Briefly, late log-phase promastigotes were cultured in 96-well plates and 2.5 × 10^6^ parasites were added per well. Formulations were diluted in deionized water up to a AmB concentration of 1 mg/mL. All samples were added in triplicate. After 48 h of incubation at 26 °C, a resazurin solution (20 μL, 2.5 mM) was added to each well and, 3 h later, the fluorescence intensity (535 nm excitation wavelength, 590 emission wavelength) was measured using a Tecan Infinite 200 fluorimeter (Tecan Group Ltd., Männedorf, Switzerland). Data analysis was performed using GraphPad Prism software version 7.02 (GraphPad Software Inc., San Diego, CA, USA) to calculate growth inhibition. The efficacy of each formulation was expressed as the IC_50_ (concentration needed to inhibit growth in 50% of the parasites).^33^

#### 2.2.6 In Vitro Cytotoxicity in J774 Macrophages

J774 macrophages were cultured in RPMI-1640 medium supplemented with FBS, penicillin (100 IU/mL), and streptomycin (100 μg/mL). Cells were then maintained at 37 °C in a humidified environment (5% CO_2_). Macrophages were placed in 96-well plates (5 × 10^4^ cells/well) with 100 μL of RPMI-1640 medium and incubated for 24 h at 30 °C, 5% CO_2_. Later, the medium was discarded; it was replaced with 200 μL of formulation and incubated again for 24 h. After that, resazurin (20 μL, 1 mM) was added, and the plates were incubated for another 3 h. Then, the absorbance was measured at 590 and 595 nm. All samples and controls (drugs dissolved in DMSO) were tested in triplicate. The safety of each formulation was assessed by the calculation of the CC_50_ (concentration needed to produce cytotoxicity in 50% of macrophages). Selectivity index (SI) was used to evaluate the relative toxicity to healthy macrophages compared to the efficacy against Leishmania. SI was calculated as follows (eq 4)­

4 $$SI=\frac{{CC}_{50}}{{IC}_{50}}$$

where CC_50_ was the concentration needed to produce cytotoxicity in 50% of healthy macrophages and IC_50_ was the concentration needed to inhibit growth in 50% of parasites.^33^

#### 2.2.7 Ex Vivo Hemolysis in Red Blood Cells

The hemolytic toxicity assay was conducted using a modified version of a previously described method.^42,43^ Blood was collected from a healthy human donor in lithium–heparin-coated Vacutainer tubes (Becton, Dickinson and Co., New Jersey, USA) to prevent coagulation. The blood was then centrifuged at 1000 g for 5 min to separate the red blood cells (RBCs) from the plasma. After the plasma was removed, the RBCs were washed multiple times with a 150 mM NaCl solution, with centrifugation repeated between washes. The washed RBCs were then diluted to a 4% (v/v) concentration using phosphate buffer (pH 7.4).

For the assay, granule dispersions were prepared in water at AmB concentrations ranging from 0.78 to 100 μg/mL. These dispersions were combined with the diluted RBCs in a 1:1 v/v ratio in 96-well plates, with all tests performed in triplicate. Positive control wells containing 20% Triton X-100 (Merck KGaA, Darmstadt, Germany) and negative control wells with PBS (pH 7.4) were included. The plates were incubated at 37 °C for 1 h using an orbital rocker, then centrifuged at 500*g* for 5 min to pellet intact RBCs. The supernatant was transferred to a new clear plate, and the absorbance was measured at 570 nm using a BioTek ELx808 UV-plate reader (BioTek Instruments Inc., Vermont, USA) to quantify released hemoglobin, indicating cell lysis.

The hemolytic toxicity was then calculated using the following equation (eq 5)­

5 $$hemolysis\;\left(\%\right)=\frac{ABS\left(sample\right)-ABS\left(PBS\right)}{ABS\left(TritonX\right)}\times 100$$

#### 2.2.8 In Vivo Efficacy Studies against Leishmania

L. infantum parasites (MHOM/BR/72/46 strain) were grown in M199 medium (Sigma-Aldrich, Darmstadt, Germany) supplemented with 10% fetal calf serum, 50,000 IU/mL penicillin, and 50 μg/mL streptomycin, at 25 °C. Stationary phase promastigotes were used throughout the entire study.

Female golden hamsters (Mesocricetus auratus) (8 weeks old) were obtained from the Medical School of the University of São Paulo, Brazil. This study was carried out in strict accordance with the recommendations detailed in the Guide for the Care and Use of Laboratory Animals of the Brazilian National Council of Animal Experimentation (http://www.cobea.org.br). The protocol was approved by the Ethics Committee of Animal Experiments of the Institutional Committee of Animal Care and Use at the Medical School of São Paulo University (numbers 056/16 and 344A). For all experimental procedures, the animals were anaesthetised with thiopental (1 mg/200 μL).

25 animals were intraperitoneally infected with 2 × 10^7^ promastigote forms of L. infantum. 60 days after infection, L. infantum infected hamsters were divided into five groups: group 1 (*n* = 5) was orally treated with the formulation consisting of the uncoated AmB core (F1), group 2 (*n* = 5) was orally treated with the combined formulation consisting of AmB and MLT (F2), group 3 (*n* = 5) was intraperitoneally treated with AmB-deoxycholate (AmB control), group 4 (*n* = 5) was orally treated with MLT (MLT control), and group 5 (*n* = 5) was orally treated with vehicle solution (PBS control).^44,45^ F1 and F2 were administered after being previously dispersed in water to achieve an AmB concentration of 1 mg/mL. All treatments were administered daily (at a fixed AmB dose of 5 mg/kg and a MLT dose of 2.33 mg/kg) once a day over the course of 7 consecutive days. Animals were weighted before and after the 7 day treatment to monitor any variations in weight that could indicate gastrointestinal toxicity. Weight differences were calculated as follows (eq 6)­

6 weightdifference(g)=weightaftertreatment(g)−weightbeforetreatment(g)

Seven days after the last dose, animals were sacrificed in a CO_2_ chamber, and the spleen and liver were collected to quantify tissue parasitism by limiting-dilution assay.^46^

Briefly, fragments of the spleen and liver from the different groups were aseptically collected, weighed, and homogenized in M199 medium. The suspensions of organs were subjected to 12 serial dilutions with four replicate wells. The number of viable parasites was determined based on the highest dilution rate where promastigote forms could be observed after 15 days of cultivation at 25 °C.

#### 2.2.9 Statistical Analysis

Minitab 16 (Minitab Inc., Coventry, United Kingdom) was utilized to perform one-way ANOVA. Tukey’s test was used to establish a comparison among formulations and find statistically significant differences between groups. *P*-value <0.05 between groups was considered a statistically significant difference.

## 3 Results

### 3.1 Physicochemical Characterization of Amphotericin B–Miltefosine Granules

#### 3.1.1 Amphotericin B Content

The AmB content in the uncoated granules was 21.05 ± 1.43% (Table 3). Following the coating process, a reduction in AmB loading was observed, with values around 18%, which was attributed to the dilution effect resulting from the addition of the coating layer, which increased the overall mass of the granules without contributing additional AmB.

**Table 3:** Physicochemical Properties of Coated Granules (*n* = 3)^a^

formulation	drug in the core	composition (binding agent)	drug in the coating layer	AmB content (%)	water content (%)	*D*_50_ (μm)	thickness of coating layer (μm)	surface area (m^2^/g)
F1	AmB			21.05 ± 1.43	7.95 ± 0.03	674.0 ± 11.9		2.511 ± 0.008
F2	AmB	Soluplus	MLT	18.04 ± 1.73*	3.48 ± 0.03	1091.5 ± 5.6	208.75 ± 8.75	1.140 ± 0.007
F3	AmB	HPMC–AS Poloxamer 188	MLT	18.21 ± 1.81*	2.34 ± 0.04	903.6 ± 7.5	114.8 ± 9.70	0.564 ± 0.002

a Key: statistically significant differences between F1 and the other two formulations were represented by * (*p* < 0.05, one-way ANOVA post-hoc test).

#### 3.1.2 Particle Size Distribution, Surface Area, and Morphology

The *D*_50_ of uncoated granules was 674 ± 12 μm. After coating, the radius of the granules increased by approximately 200 μm when using Soluplus and by about 115 μm with HPMC–AS and Poloxamer 188. These values represent the thickness of the coating layer (Table 2). Conversely, the surface area decreased 2–4-fold from uncoated to coated granules as the coating layer acted as a sealing protective layer (Table 2) which was aligned with the reduction in the water content from ∼8% to below 4% (Table 2) in the coated granules.

SEM micrographs revealed that uncoated granules were quasi-spherical, ranging from 500 μm in size to 800 μm in size (Figure 1a,b). Their surface comprised multiple small crystals (1–20 μm), explaining their greater surface area. In contrast, coated formulations showed a smoother surface (Figure 1c–f). Cross-sectional images of halved granules showed the porous core structure and the thickness of the coating layer (Figure 1d,f).

**Figure 1:**
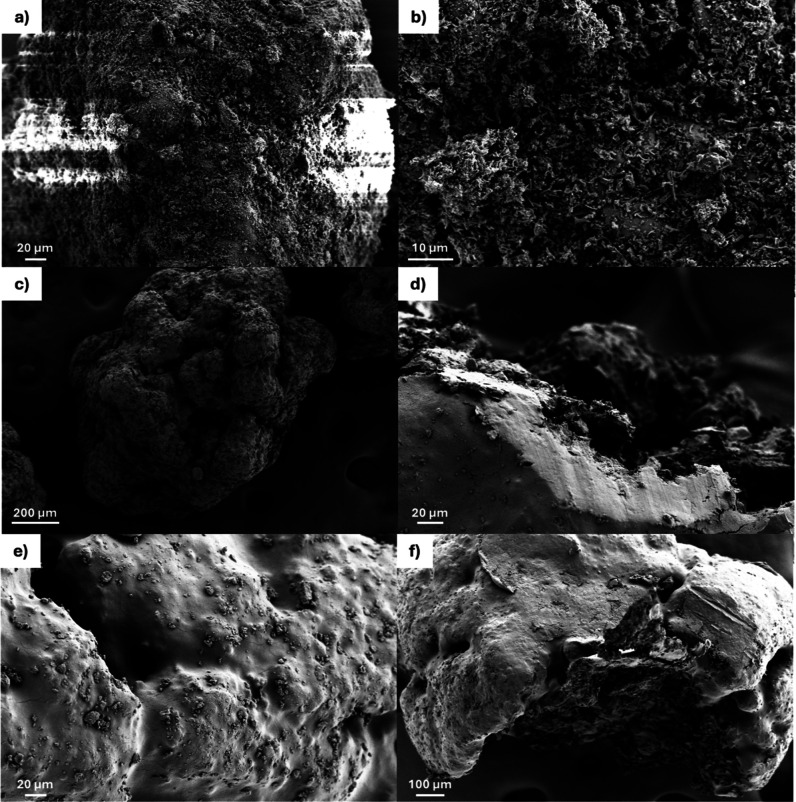
SEM micrographs of F1 (a,b), F2 (c,d), and F3 (e,f) showing the morphology of the core and the coating layer of each formulation.

#### 3.1.3 Modulated Temperature-Differential Scanning Calorimetry

DSC thermogram of AmB shows a notable broad endotherm at 148.1 °C, which is consistent with literature reports of the thermal behavior of AmB, possibly indicating melting followed by degradation (Figure 2a). DSC thermogram of MLT monohydrate shows a dehydration endotherm with an onset temperature of 89.9 °C which matches with previous reports.^47^ The subtle event at 51.9 °C is attributed to a solid–solid transition or the melting of a minor impurity (Figure 2a). The melting event of MLT was not recorded as occurs at higher temperatures being described as melt-decomposition endotherm with onset at 265.4 °C.^47^ The formulations demonstrated similar thermal profiles to individual components but with lower heat flows, which were attributed to the dilution factor with the excipients as well as the amorphization occurring during the spray-coating process.

**Figure 2:**
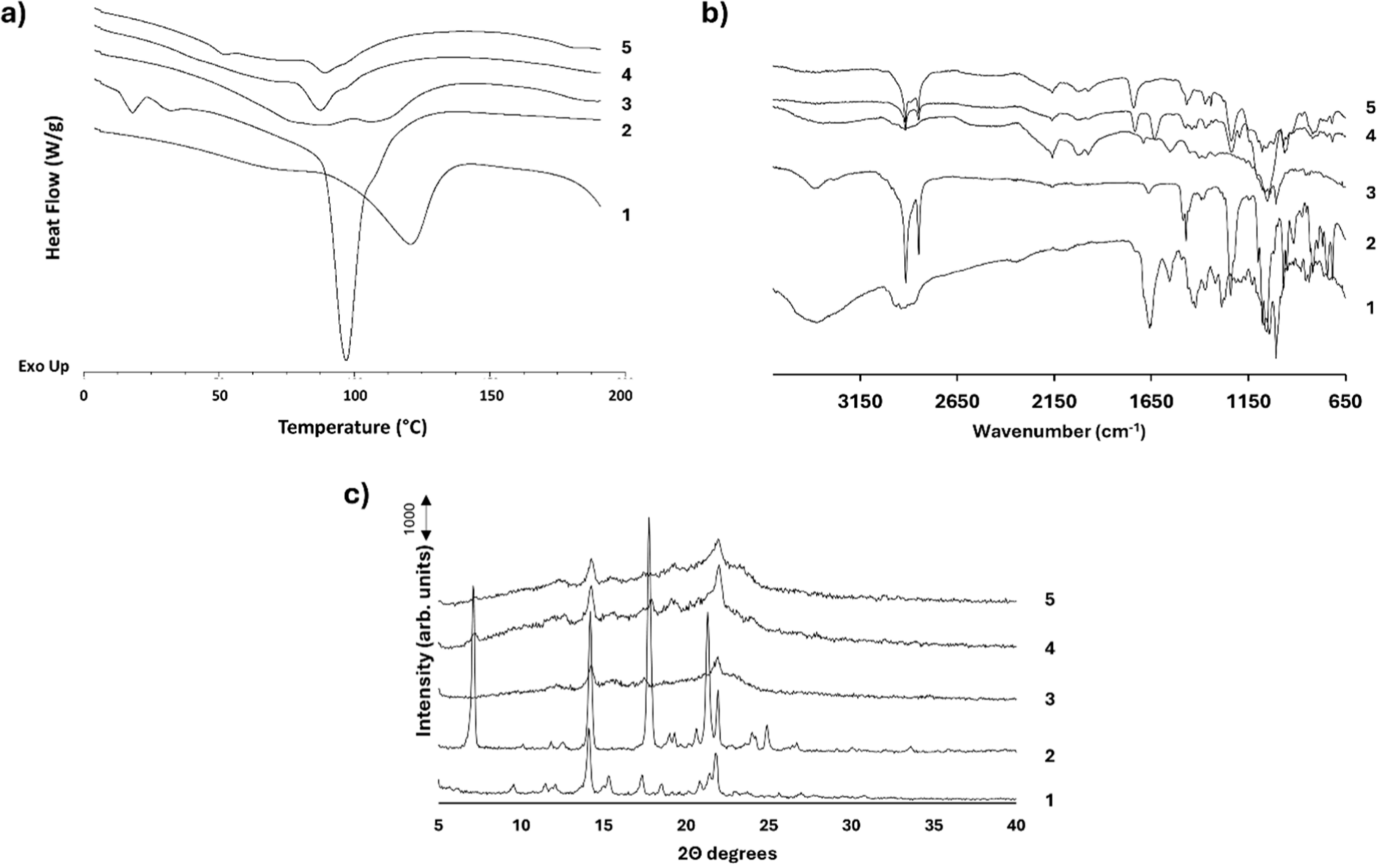
Physicochemical characterization of FDC granules: (a) DSC thermograms; (b) FTIR spectra; and (c) PXRD patterns. Key: AmB raw material (1), MLT raw material (2), F1 (3), F2 (4), and F3 (5).

#### 3.1.4 Fourier-Transform Infrared Spectroscopy

The FTIR spectral analysis of AmB exhibited a broad peak between 3481 and 3294 cm^–1^, indicative of O–H or N–H stretching, along with a weak, but characteristic peak at 1556 cm^–1^, likely due to C=C stretching in the conjugated double bonds in the hydrophilic chain of AmB. In contrast, MLT displayed a sharp C–H stretching peak at 2914 cm^–1^ (Figure 2b). F1 retained similarities to the AmB spectrum, with some modifications in peak intensities but no shift in the characteristic peak at 1556 cm^–1^. Coated formulations both demonstrated a merger of AmB and MLT spectral features, with subtle differences in the fingerprints in the 1500–1000 cm^–1^ region (Figure 2b). Interestingly, the AmB peak at 1556 cm^–1^ disappeared for both F2 and F3, likely due to the interaction between AmB and the coating materials. To sum up, all formulations exhibited some degree of peak broadening and intensity changes compared with the raw materials.

#### 3.1.5 Powder X-ray Diffraction

AmB exhibited characteristic Bragg peaks at 14.3° and 21.9° 2θ, which were also present in the uncoated granules. This indicated that AmB maintained its crystalline form after granulation. The diffraction pattern of coated granules for these peaks closely resembled those of uncoated AmB granules, but with sharper peaks, suggesting that AmB remained crystalline after spray-coating with an apparent reorganization of the crystals into a more ordered arrangement^48^ (Figure 2c). MLT, on the other hand, displayed distinct Bragg peaks at 7.1°, 14.5°, 17.7°, and 21.9° 2θ. However, in the F3 formulation coated with HPMC-AS/Poloxamer 188, only AmB-associated Bragg peaks were observed (Figure 2c). The absence of MLT peaks in this formulation suggested that MLT oxidation transitioned to an amorphous state during the coating process.

#### 3.1.6 Dynamic Vapor Sorption

DVS analysis revealed distinct moisture uptake patterns for uncoated and coated granules (Figure 3a). The uncoated formulation (F1) exhibited the highest water uptake, reaching approximately 20% at 90% RH, displaying a more pronounced hysteresis. In contrast, the coated formulations F2 and F3 showed a lower water uptake, with maximum values of 17.5% and 14.8%, respectively. Notably, all formulations maintained consistent mass across two sorption–desorption cycles, indicating stability against recrystallization. This stability was further corroborated by PXRD analyses conducted post-DVS, which revealed no alterations in the diffraction patterns of any formulation (Figure 3b).

**Figure 3:**
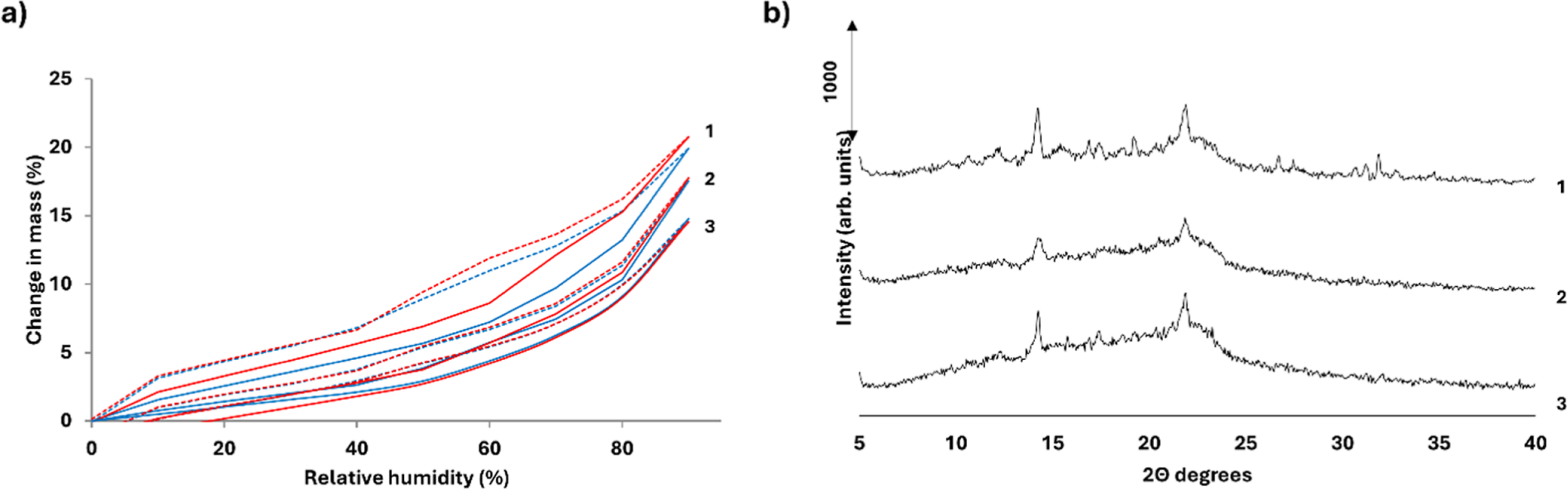
DVS sorption (solid lines) and desorption (dash lines) isotherms of first (blue lines) and second cycles (red lines) (a) and post-DVS PXRD analyses (b). Key: F1 (1), F2 (2), and F3 (3).

### 3.2 Dissolution Studies

Figure 4 illustrates the dissolution profiles of AmB across the three formulations. The uncoated AmB core (F1) demonstrated pH-dependent release kinetics. At pH 1.2, less than 1% of AmB was released within the first 30 min. However, upon increasing the pH to 6.8, a rapid release was triggered, resulting in a complete dissolution after 180 min. Dissolution data modeling revealed that F1 followed Korsmeyer–Peppas kinetics with Fickian diffusion (release exponent, *n* < 0.43) (Table 4). Notably, the granules maintained their structural integrity for 2–3 h during the dissolution study, while their density allows them to float for the whole duration of the experiment. The polymer coating in formulations F2 and F3 played a critical role in modulating the release of AmB. The coating materials (Soluplus in F2 and HPMC-AS/Poloxamer 188 in F3) served as diffusion barriers as these formulations exhibited significantly slower dissolution rates compared to F1 and exhibited approximately 50% release rate after 24 h (Figure 4). Both coated formulations also followed Korsmeyer–Peppas kinetics, but with distinct mechanisms: F2 displayed Fickian diffusion (*n* < 0.43), while F3 showed an anomalous mechanism combining diffusion and erosion (0.43 < *n* < 0.85) (Table 4).

**Figure 4:**
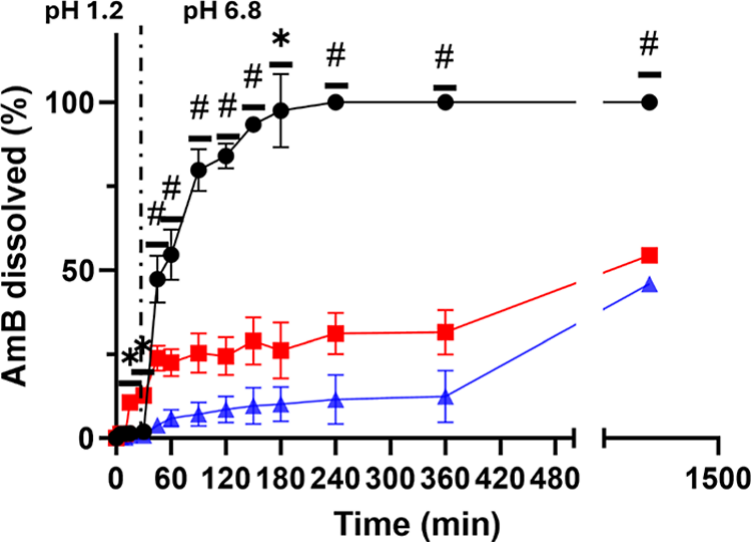
Cumulative drug release profile for AmB. Key: F1 (black circles), F2 (red squares), and F3 (blue triangles). Statistically significant differences between F1 and the other two formulations were represented by *, while # showed statistically significant differences among the three formulations (*p* < 0.05, one-way ANOVA posthoc test).

**Table 4:** Drug Release Kinetics from FDC-Coated Granules

formulation	zero order (*R* ^2^)	first order (*R* ^2^)	Higuchi (*R* ^2^)	Korsmeyer–Peppas (*R* ^2^)	Hixson–Crowell (*R* ^2^)
F1	0.8808	0.9658	0.9698	0.9958 (*n* = 0.2940)	0.9546
F2	0.6667	0.7305	0.8098	0.9763 (*n* = 0.279)	0.7083
F3	0.9680	0.9671	0.9091	0.9724 (*n* = 0.821)	0.9685

### 3.3 Accelerated Predictive Stability Studies

Formulations F1 and F2 were evaluated for long-term stability and APS. F3 was discarded bearing in mind the slow-release kinetics in physiological media. Long-term stability studies revealed that all formulations maintained chemical stability for at least 1 year under refrigerated conditions and at room temperature in desiccated environments, with the drug content remaining above 90%. Humidity significantly impacted the chemical stability of AmB in the uncoated formulation (F1), as evidenced by a greater *B* term of 0.007. In contrast, F2 showed no susceptibility to humidity, suggesting that its coating effectively shielded AmB from moisture. Table 5 presents the degradation kinetics and activation energies for AmB, which followed a second-order kinetic model in both formulations.

**Table 5:** Degradation Models of AmB at the Tested Conditions of Temperature and RH^a^

formulation	best-fitting model	*E*_a_ (kcal/mol)	*B* term	predicted stability at 4 °C and 10% RH (years)	experimental drug content after 1 year at 4 °C and 10% RH (%)	experimental drug content after 1 year at 25 °C and 10% RH (%)
F1	second order	15.049 ± 7.213	0.007 ± 0.002	0.977 ± 0.039	93.607 ± 1.312	91.153 ± 0.464
F2	second order	19.146 ± 3.644	0.000 ± 0.000	1.144 ± 0.039	94.187 ± 1.653	91.793 ± 1.666

a Key: *E*_a_ (activation energy); *B* term (moisture sensitive factor).

The prediction models closely aligned with the long-term experimental results, demonstrating good overall chemical stability for the formulations when stored under refrigerated or room temperature desiccated conditions for a year.

### 3.4 Antileishmanial Activity and Cytotoxicity of Fixed-Dose Combination Granules

The *in vitro* antileishmanial activity of FDC granules is illustrated in Table 5. L. donovani and L. infantum were selected as these parasites are the etiological agents responsible for VL. All FDC formulations exhibited a greater activity (3–9-fold) against L. infantum over L. donovani. Notably, uncoated AmB granules were the most active formulation against both Leishmania species, while dual drug-coated formulations exhibited higher values of IC_50_, which aligned with the results from AmB dissolution. This can be attributed to the slower drug release from coated granules compared with the uncoated AmB formulation. AmB–DMSO controls were still more active than any of the three tested formulations.

Regarding toxicity, it was observed that FDC formulations assayed on J774 macrophages and RBCs exhibited a safer profile (SI > 10) than AmB dissolved in DMSO, specially formulations F1 and F3 for macrophages as well as F1 and F2 for RBCs (Table 6). F1 demonstrated the most favorable efficacy/safety balance among the formulations, as evidenced by its SI values. For L. donovani, the SI value was 148.15 ± 1.35, while for L. infantum, it reached 444.44 ± 4.51. These calculations were based on the maximum tested concentration of 200 μg/mL. Notably, F1 showed no cytotoxicity for macrophages at this concentration, suggesting that the actual SI values are even higher than reported in Table 6.

**Table 6:** *In Vitro* Efficacy and Toxicity of FDC Granules^a^

formulation		AmB–DMSO	F1	F2	F3
L. donovani	IC_50_ (μM)	0.23 ± 0.01	1.35 ± 0.15	4.65 ± 0.19	4.59 ± 0.20
	SI	51.48 ± 10.09	≥148.15 ± 1.35*	3.20 ± 2.38	17.94 ± 2.49
L. infantum	IC_50_ (μM)	0.09 ± 0.01	0.45 ± 0.10	1.16 ± 1.26	0.48 ± 0.05
	SI	131.56 ± 20.18	≥444.44 ± 4.51*	12.85 ± 2.35	171.50 ± 2.41
CC_50_ (μM)		11.84 ± 4.03	N/A	14.91 ± 4.57	82.32 ± 4.77
HC_50_ (μM)		37.14 ± 2.81	25.61 ± 10.15	43.17 ± 0.10	7.23 ± 1.18

a Key: IC_50_ (concentration needed to inhibit growth in 50% of parasites), SI, * (SI calculated at 200 μg/mL), CC_50_ (concentration needed to produce cytotoxicity in 50% of healthy macrophages), N/A (F1 did not exhibit any cytotoxicity at the highest tested concentration of 200 μg/mL), and HC_50_ (concentration needed to produce hemolysis in 50% of RBCs).

### 3.5 In Vivo Efficacy Studies against Leishmaniasis

F1 (uncoated AmB granules) and F2 (coated AmB granules) were administered orally *in vivo* in golden hamsters infected with L. infantum at a fixed dose of 5 mg/kg once a day over 7 consecutive days. The same dose regime was followed to intraperitoneally administer an AmB control and orally administer both a MLT and PBS control. The parasite burden in the liver was reduced by 41, 81, and 65% after the administration of AmB control, F1, and F2, respectively, while in the spleen, it was reduced by ∼65% for both tested formulations and by 79% for the AmB control. However, the animals treated with oral MLT only showed a significant decrease in parasite load in the spleen (43%) (Figure 5a).

**Figure 5:**
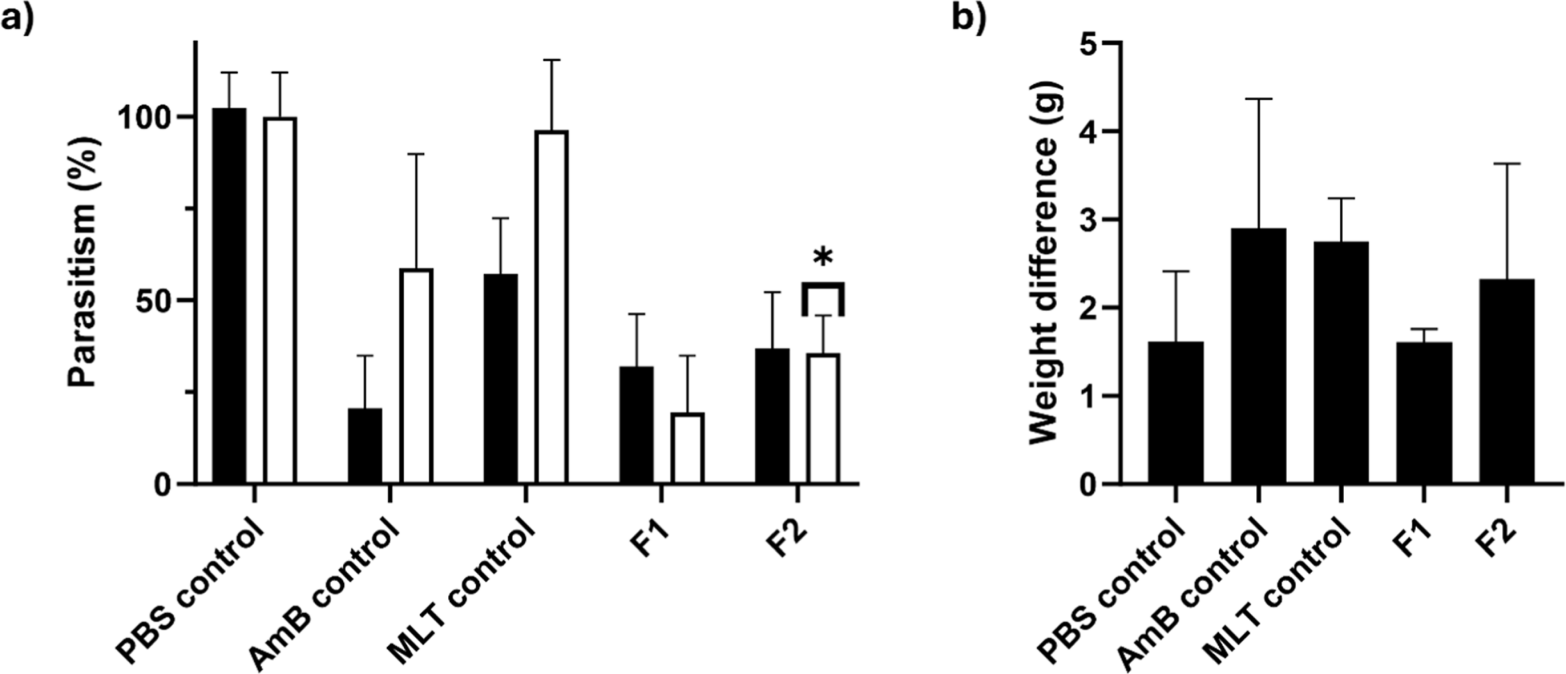
*In vivo* evaluation of FDC granules in L. infantum infected golden hamsters: (a) Cumulative parasite burden (L. infantum) in the spleen (black) and liver (white) after a once-daily 7 day treatment course with AmB control (5 mg/kg), MLT control (2.33 mg/kg), F1 (5 mg/kg), and F2 (5 mg AmB/kg and 2.33 mg MLT/kg); (b) body weight change in golden hamster before and after the 7 day treatment course. Statistically significant difference between the formulation groups and the MLT control group is represented by * (*p* < 0.05, one-way ANOVA posthoc test). No significant difference was observed between the formulation groups and the AmB control.

In addition, animals gained weight during the whole treatment, indicating good gastrointestinal tolerance and absence of acute gastrointestinal toxicity (Figure 5b).^49^

## 4 Discussion

The development of oral therapies for the treatment of VL remains challenging due to the poor aqueous solubility of most antileishmanial drugs. To the best of our knowledge, this is the first report of an orally fixed-dose formulation combining AmB with MLT. In this work, the *in vitro* and *in vivo* performance of uncoated and coated AmB–MLT granules was explored to understand the impact of a coating sealing layer on AmB granules to protect them from moisture. The morphology of the granules was significantly transformed after coating from a rough surface to a smooth surface. The sealing effect was corroborated by DVS studies indicating a lower hygroscopicity of coated granules which was aligned with greater chemical stability but resulted in a detriment in the release kinetics that impacted the final *in vivo* performance.

Drug release studies provided valuable insights into the behavior of the formulations. F1 demonstrated the fastest AmB release with a pH-dependent dissolution profile, with minimal release occurring under acidic conditions. This characteristic is particularly advantageous as it could prevent premature drug release in the acidic environment of the stomach where AmB degrades faster, instead favoring release in the intestine, where absorption is more efficient. The coated formulations, F2 and F3, exhibited slower AmB release rates due to the barrier effect of the coating layer. All formulations followed Korsmeyer–Peppas release kinetics, though with varying mechanisms depending on their composition. While F1 and F2 fitted to a Fickian diffusion (*n* < 0.43), F3 showed an anomalous mechanism combining diffusion and erosion (0.43 < *n* < 0.85). The latter is usual in oral formulations containing cellulose derivatives, such as HPMC, so it could be attributed to the polymers employed for coating, while Fickian diffusion could be explained by the quasi-spherical shape of the granules, which was maintained during the dissolution study for at least 2 h. As the shape of the granules influences the surface area exposed to the dissolution medium and how the drug molecules move out of the matrix, a quasi-spherical shape has a relatively constant surface area during dissolution, meaning that the rate at which the drug is released remains steady over time.^50^ Nevertheless, MLT release was not studied due to its high aqueous solubility (≥2.5 mg/mL) and the lack of strong chromophores in its chemical structure, which makes ultraviolet and fluorescence detection very difficult.^51^

Stability studies demonstrated that all formulations remained chemically stable for at least 1 year when refrigerated and at room temperature under desiccated conditions. RH played a crucial role in AmB stability, particularly affecting F1 due to the lack of a protective coating layer. The coating in F2 served as an effective moisture barrier, enhancing its stability. In both F1 and F2, AmB degradation followed second-order kinetics. This suggests that the degradation process is more complex, and the degradation rate does not depend linearly on concentration. A second-order model implies that interactions between AmB molecules, between AmB and MLT, or between AmB and excipients may play a significant role in the degradation process, rather than the sole influence of the concentration of AmB.^52^ The coating layer did not modify the degradation kinetics of AmB, unlike in the previously developed formulation containing AmB in the core and itraconazole in the coating layer, in which AmB followed an Avrami degradation model.^9^

The combination of MLT alongside intravenous liposomal AmB in HIV-coinfected VL patients in East Africa significantly improved cure rates and reduced failure rates for both primary VL and VL relapse compared to high-dose AmBisome monotherapy.^53^ This shows immense potential for combining AmB and MLT within the same oral solid dosage form to facilitate regimens and enhance patient compliance and clinical performance. To maintain the physicochemical compatibility between both drugs, they were contained in different sections of the granules, AmB in the core, and MLT in the coating layer, minimizing physicochemical interactions.

The formulations showed significant *in vitro* antiparasitic activity against both L. donovani and L. infantum *,* with F1 demonstrating the highest efficacy, which is aligned with its fastest release. Based on their *in vitro* performance, F1 (uncoated AmB granules) and F2 (coated AmB–MLT with Soluplus) were selected for further *in vivo* testing, revealing that both formulations significantly reduced the presence of L. infantum parasites in the spleen and liver of golden hamsters compared to the control group. This indicated drug accumulation in both tissues to elicit the pharmacological effect observed. The combination of AmB and MLT did not result in higher efficacy compared to the uncoated granules which can be attributed to the partial release of AmB from the core. However, we hypothesize that upon dissolution in the gastrointestinal tract, MLT could enhance AmB solubilization triggering into micelles and promoting absorption through the Peyer’s patches. Serrano et al. showed that AmB encapsulation in modified chitosan nanoparticles significantly enhanced oral bioavailability by up to 24% through the gut-associated lymphoid tissue via the lymphatic vessels to the systemic circulation, which explains the drug accumulation in the spleen and the liver.^17^ Additionally, the increase in body weight is an encouraging insight into the overall tolerability of these FDC formulations, which is related to optimal gastrointestinal tolerability, although toxicity studies should be carried out to assess the safety of the formulation and to confirm that the increase in body weight is not related to ascites due to liver failure.

Even though the coating layer protected AmB from degradation, further development should aim for a faster release at the intestinal pH to enhance oral bioavailability and then *in vivo* efficacy as the slow release of AmB might cause a lack of absorption of the drug before being excreted. Formulating both drugs in the core could be advantageous to increase the MLT dose in the formulation and also to diminish the thickness of the coating layer to ensure a faster release. A previous study in our group demonstrated that combining AmB with itraconazole with a similar oral drug delivery strategy elicited a pharmacological effect leading to drug concentrations above the minimum inhibitory concentration in liver and spleen for AmB.^9^ In this case, MLT is more water-soluble than itraconazol, so we expect an enhanced oral absorption of AmB. However, further *in vivo* pharmacokinetic studies need to be carried out to confirm this hypothesis.

In terms of chemical stability and *in vivo* efficacy, it has been acknowledged that the coated formulations did not outperform F1. However, the inclusion of the coating provided a mechanism for sustained release of AmB, which might be beneficial for maintaining therapeutic drug levels over time, potentially improving the efficacy and safety of the treatment. Although the dissolution rates obtained *in vitro* were slower, this could help mitigate the adverse effects associated with the rapid release of AmB, improving overall patient compliance by reducing the frequency of dosing.

These results collectively suggested that AmB-MLT FDC granules represent a valuable approach for treating VL orally. The ability to significantly reduce parasite burden combined with good gastrointestinal tolerability indicates that the oral treatment could be prolonged over 7 days, aiming for a better pharmacological response. The oral administration of these formulations thus offers a convenient alternative to traditional parenteral AmB formulations, potentially improving patient compliance and treatment accessibility.

## 5 Conclusions

The development of oral AmB–MTL FDC granules represents a significant advancement in the treatment of VL. This approach offers several advantages over traditional intravenous AmB administration. First, it provides a more patient-friendly treatment option that eliminates the need for hospitalization, a crucial benefit in developing countries where healthcare resources are often limited. The formulations demonstrated remarkable stability for at least 1 year and showed promising results against two Leishmania species. In vivo studies rendered encouraging results, with a 65–80% reduction in parasite burden in the liver and spleen, which are the primary sites of accumulation in VL. Furthermore, the increase in body weight after the 7 day treatment course suggests that the formulation is well-tolerated for oral administration with no apparent gastrointestinal toxicity. These findings collectively indicate that oral AmB–MLT FDC granules could be a game-changing approach in clinical practice for VL treatment, offering improved patient compliance, accessibility, and potentially better therapeutic outcomes.
